# What can ecological studies tell us about death?

**DOI:** 10.1186/s13584-017-0176-x

**Published:** 2017-10-02

**Authors:** Yehuda Neumark

**Affiliations:** Braun School of Public Health and Community Medicine, Hebrew University of Jerusalem, P.O. Box 1227, 99112102 Jerusalem, Israel

**Keywords:** Ecological fallacy, Ecological studies, Epidemiology, Mortality, Study design

## Abstract

Using an ecological study design, Gordon et al. (Isr J Health Policy Res 6:39, 2017) demonstrate variations in mortality patterns across districts and sub-districts of Israel during 2008–2013.

Unlike other epidemiological study designs, the units of analysis in ecological studies are groups of people, often defined geographically, and the exposures and outcomes are aggregated, and often known only at the population-level.

The ecologic study has several appealing characteristics (such as reliance on public-domain anonymous data) alongside a number of important potential limitations including the often mentioned ‘ecological fallacy’. Advantages and disadvantages of the ecological design are described briefly below.

## Main text

"The aim of epidemiology is to decipher nature with respect to human health and disease, and no one should underestimate the complexities of epidemiological research" [1]. To achieve this lofty and complex goal, the epidemiologic investigative toolbox contains various study methodologies including individual-based experimental designs (e.g., randomized controlled trial), individual-based observational designs (e.g., prospective cohort study, retrospective case-control study) and group-based, or ecological studies. As articulated by Susser [2], the aim of ecological analysis is "to study health in an environmental context… to understand how context affects the health of persons and groups through selection, distribution, interaction adaptation, and other responses".

In an ecological study, the units of analysis and comparison are groups of people often defined geographically (such as an administrative region or an entire country), and the exposures and outcomes are aggregated and known at the population-level only. Morgenstern [3] categorized ecologic studies into ‘exploratory’ studies that compare rates of disease or other outcomes across groups in a descriptive fashion and do not attempt to correlate these rates with exposure data, and ‘multiple-group comparison’ studies that explore associations between average exposure levels and rates of the outcome across groups.^1^

In this journal, Gordon et al. [4] employ an ecological design to demonstrate variations in mortality patterns across districts and sub-districts of Israel during the five-year period of 2008–2013. Standardized mortality ratios (SMRs) reveal a 25% excess of kidney disease related deaths in the Haifa district, for example, while the risk of death from influenza/pneumonia is 25% lower there than the national average. Some attenuation of regional differences is noted upon adjusting for “ethnicity” (Arabs; Jews by continent of birth) leading the authors to conclude that "factors associated with ethnicity may affect mortality more than regional factors". The authors also correlate SMRs with selected district-level socio-economic characteristics, and demonstrate significant inverse correlations (−0.63–0.71) between (sub)district-average years of education and all-cause mortality in males and females, cancer-related death and diabetes-related death. Significant negative correlations (−0.52–0.57) are also found with the percent of district residents who purchased supplementary health insurance and cancer, heart and diabetes related mortality. The (sub)district-level prevalence of smoking correlates positively with SMRs for diabetes-related mortality and all-cause mortality in males (0.59) and females (0.63).

The ecologic study has several appealing characteristics, primary of which is the reliance on anonymous (often public domain) data that cover large geographical areas, even nationwide. This is particularly pertinent when individual-level data is lacking or not readily available, and even when such data can be collected, the use of secondary data sources has an obvious advantage in terms of cost and time.^2^

Another advantage of using aggregated data is the avoidance of measurement error of individual-level exposures. Valid information about personal consumption of alcoholic beverages, for example, may be difficult to obtain, whereas aggregated data (e.g., from alcohol tax revenue records) may provide more accurate information. This is certainly the case with air pollution exposures that are difficult to ascertain accurately at the individual level, and are more feasibly measured via ambient air monitoring.

Ecological studies have been employed extensively to assess and explain regional variations in mortality, such as Lavados et al. [5] who found significant differences in age-adjusted stroke-related mortality across 13 administrative regions in Chile. Merging data from the national death registry with individual-level data aggregated at the regional level, they concluded that socio-economic characteristics, primarily poverty, explained 34% of the stroke-related mortality variance across regions, and cardiovascular risk factors (i.e., diabetes, sedentarism and overweight) explained an additional 26% of the variability.

Ecological analyses have also been undertaken to examine mortality patterns in Israel. Examining sub-district mortality variations using data from 1987 to 1994, Ginsberg et al. [6] found elevated standardized mortality ratios (SMR) adjusted for age, sex and ethnicity in the Haifa district for all cardiovascular diseases, liver disease, motor vehicle accidents and lung cancer. The authors concluded that the observed regional mortality differences "may be due to socioeconomic, nutritional, environmental, occupational, or health care factors". In 2008, responding to public concern about exposure to industrial park emissions in the south of Israel, Karakis et al. [7] used an ecological approach to demonstrate a correlation between mortality rates (1995–2001) and residential proximity to the industrial park among the Bedouin population. More recently, in this journal, Goldberger & Haklai [8] demonstrated steady national declines in age-adjusted rates of “amenable deaths” (i.e., deaths that could be prevented by effective health care), although regional variations persist. For example, for the period 2007–2009, compared with the national average, amenable mortality was 14% higher in the southern district among females and 18% higher in males.

Gordon et al. note several potential limitations inherent in their study and its findings, some of which are equally applicable to other epidemiological study designs (e.g., the “survey” nature of the socio-economic and behavioral correlates and the use of continent/country of birth as an indicator for ethnicity), and some are specific, or particularly relevant to ecological studies. We will briefly focus here primarily on the latter.

The major, and most often mentioned limitation inherent in testing etiologic hypotheses through ecologic analyses is the potential of making a “mistaken assumption that a statistical association observed between two group-level variables is equal to the association between the corresponding variables at the individual level” (Gail & Benichou, 2001). This potential bias is known as ‘ecological fallacy’ (sometimes referred to as ‘aggregation bias’ or ‘cross-level bias’.

The discordance, or at least lack of necessary concordance, between individual-level and ecological-level correlations, was first described mathematically by Robinson [9] in his seminal paper on ecological correlations. Robinson used a classic 2*2 table approach to elegantly illustrate that a given set of marginal frequencies (i.e., the disease/death rate and the prevalence of exposure) can be generated by a large set of internal frequencies (number or rate of exposed and unexposed cases, for example). In a group-based or ecological study design, the marginal frequencies are known, while the joint distribution of exposure and outcome variables remains unknown, leading Robinson to conclude "the only reasonable assumption is that an ecological correlation is almost certainly not equal to its corresponding individual correlation" [9].

Despite Gordon et al.’s cautionary mention of ecological fallacy, a presumption of a “causal” association between education and mortality is implicit in their recommendation to "increase the education level of all sectors of the population". Their concluding recommendations of "raising the education level, reducing smoking, control of hypertension, encouraging healthy lifestyles and screening for cancer", assume that because mortality is higher in districts with larger exposure prevalence, it is those individuals with fewer years of education, smokers, and/or those who did not undergo screening, who died, or were at higher risk of death during the study period. This assumption attributes to members of the group the characteristic of the group [10]. While these are not unreasonable assumptions, they cannot be tested or supported using aggregated data.

Part of the challenge in making cross-level inferences relates to different underlying constructs being measured by the “same” variable at the individual level and the aggregate level. The classic example given for this is the effect of individual poverty and community or neighborhood poverty on health. The ILMS showed that while area-level deprivation is inversely associated with risk of death in both men and women, household deprivation relative to ones’ neighborhood (adjusted for absolute socio-economic status) is associated with mortality risk among men only [11].

Among the criteria proposed by Arsenault et al. [12] for evaluating and comparing geographical units in the context of ecological studies are theoretical considerations (e.g., biological relevance) and intra-unit homogeneity. These criteria are more likely to be attained when the geographical units being analyzed are defined by their relevance to the research question, rather than “administrative” units, as is the case here. Causal group effects, suggests Firebaugh [13], "seem most likely in groups where group members interact and share relevant life experiences; hence, the macroproperties of 'natural' groups (neighborhoods, for example) seem more likely to have causal effects than the macroproperties of arbitrarily-created regions (census tracts, for example)". Large intra-area heterogeneity with regard to exposure variables, and variation in the size of the geographic units being compared, increase the likelihood of bias from aggregated data being used as an approximation for individual-level data [12, 14, 3].^3^ Gordon and colleagues acknowledge the possibility that "because variations in income within sub-districts are great, the average income does not reflect the true income distribution". Noting the large intra-unit population heterogeneity and the large variation in sub-district size, the authors caution that "Drawing conclusions from comparisons between different regions in Israel may be problematic".

Another commonly-occurring shortcoming of the ecological design, more so than individual-level designs, is the issue of confounding. In ecologic analyses, confounding may exist at the individual level, the group level (e.g., geo-spatial characteristics of the grouped unit), or at both levels (cross-level confounding). Moreover, unlike at the individual level, where the risk-factor must be associated with the exposure of interest for confounding to occur, an ecologic-level confounder need not be associated with exposure in all groups [15]. Confounding in ecologic studies is further complicated by the limited ability to control for confounding effects [16] and the possibility that ecologic adjustment may accentuate the bias [17].

The complex issues of cross-level bias and confounding in ecologic analyses have received much attention in the literature and a full discussion of these and other potential limitations of ecologic studies (such as temporal ambiguity and the potential lag effect - [18, 19]; Morgenstern in Encyclopedia) can be found in reviews of this study design (e.g., [3, 10], or for the more statistically-oriented reader – [20, 16]).

The inability of ecological analyses to provide information for making inferences about etiological mechanisms at the individual level and the fear of committing ecological fallacy has led to widespread skepticism regarding the rightful place of ecological studies in the epidemiologic toolbox, and has relegated them, for the most part, to the realm of hypothesis-generating studies.

With tongue in cheek, Messerli [21] presented a strong ecologic correlation between per capita chocolate consumption levels and the number of Nobel Prize laurates in 23 countries. The author points out that "Of course, a correlation between X and Y does not prove causation but indicates that either X influences Y, Y influences X {reverse causation}, or X and Y are influenced by a common underlying mechanism {confounding}… Obviously these findings are hypothesis-generating only and will have to be tested in a prospective, randomized trial". As eminent epidemiologist Neil Pearce [22] noted: "Even when studying individual level risk factors, population level studies play an essential part in defining the most important public health problems to be tackled, and in generating hypothesis as to their potential causes".

At the same time, Loney & Nagelkerke [23] warn against ‘individualistic primacy’ - "the belief that associations on an individual level are intrinsically more truthful, i.e., better reflecting causal relationships, than those on an ecological level".^4^ In considering ecological studies, the distinction should be made between individual variables that are measured at the group-level (e.g., education, monthly income, or smoking) and “ecological” variables of the ‘human ecology’ type.^5^ Moreover, inherent conceptual differences may exist in the “same” variable measured at the individual level and at the group level (e.g., poverty), and some variables, as noted by Loney & Nagelkerke [23], only have meaning at a societal level. Research questions addressing social or ecological phenomenon, such as the investigation of the effect of macro-social policy change or broad cultural shifts on a particular aggregate health outcome, may best be served, or only served, by aggregate-level ecological studies.

Interest in ecologic analyses has been rekindled with the development of multilevel statistical modeling that simultaneously assesses individual-level and group-level characteristics and their interactions [24]. Multilevel analyses "opens up the possibilities for richer cross-level approaches that enable discerning the relative contribution of different levels to the scientific question of interest" [25], and thereby circumvents, to a large extent, the restrictive interpretation of single-level analyses.

There is a danger that the ready availability of aggregated data and the relative ease of ecological analyses, may lead to over-zealous correlation-seeking [26]. This must be tempered with hypothesis-driven inquiry that should strive to meet, or at least be guided by Sir Bradford Hill’s guidelines for causation, primarily that of “biological plausibility” [27]. As noted by Pearce [22]: "…a knowledge of appropriate methods of study design and data analysis is not a substitute for knowing how to choose the most appropriate hypothesis to study". Or, as [28] reminded us in his musings about the future of epidemiology (which we are happy to report is still very much alive and kicking, despite some dire predictions about its imminent and premature demise), "astronomy is about the cosmos and not about the telescope".

Furthermore, as noted by Saunders & Abel [26], "A simple, unadjusted, correlation of two measures at the population level has the potential for eye-catching headlines". In 2011, a public outcry flared over media reports of elevated mortality due to air pollution in the Haifa district (e.g., headlines in the December 29, 2011 online edition of Haaretz Newspaper read "Study Finds Haifa Has Highest Pollution-related Mortality Rate"). Yet, according to the official government statistics, the age-adjusted mortality rate from respiratory diseases in Haifa is 12% lower than the national average and all-cause mortality is only 2% above the national average [29]. Earlier this year, public debate and tumult re-erupted in Israel over purported deleterious health effects (including low birth weight and microcephaly) of petro-chemical and other industrial emissions in the Haifa Bay region that were widely-heralded in the press.

## Conclusions

We would be wise to heed the cautionary, yet empowering words of Wade Hampton Frost [30], often considered the father of modern epidemiology, which are relevant to all epidemiological inquiry, and perhaps more so to evidence obtained from ecological studies: “It is frequently easy to exhibit some figures which, though not really to the point, will nevertheless serve to impress an uncritical public, and the temptation may be great to give them, at least by implication, an unduly favorable interpretation. It is more difficult and more tedious to present the full argument, based on all the facts, and it is perhaps a little humiliating to admit that the statistical evidence is deficient because we have failed to collect it, but to do this is not only more scientific, it is in the end more convincing, and after all there is no free choice, because it is the only honest method, whether it be convenient or not. Finally, it is the only way of progress, for the first step towards collecting better evidence is to recognize the deficiencies of that which is at hand”.

The health of the Israeli population continues to improve, life expectancy in Israel is among the highest in the world, and while geographic and other health inequalities persist, they are narrowing in many respects (Muhsen et al., 2017). As Israel, and the rest of the world, moves forward toward ensuring healthy lives and promoting well-being for all at all ages (Sustainable Development Goal #3) we need to be mindful of, but not daunted by, the challenges of collecting accurate and pertinent data and drawing valid inferences from the data at hand.

## Abbreviations

SMR: Standardized Mortality Ratio

## Footnotes

1 Morgenstern [3] also described ecologic studies that correlate changes in exposure levels with changes in outcome rates in a single group (‘time-trend’ studies), and across multiple groups (‘mixed’ studies).

2 In Israel, thanks to the unique identification number assigned to every resident, newborn and immigrant, detailed mortality patterns can be ascertained and investigated by linking vital status data from the National Population Registry with individual-level data sources. Linking vital status data with national census data, the Israel Longitudinal Mortality Study (ILMS) demonstrated, for example, widening educational differentials in mortality over time [31] and a protective effect of marriage on mortality [32].

3 The bias, resulting from the imposition of “artificial” boundaries on continuous geo-spatial units, was first described by Gehlke and Biehl [33] and is often referred to as the ‘modifiable areal unit problem’ [34].

4 The lesser-known term ‘individualistic fallacy’ (also known as “atomistic fallacy”), has been used to describe “the failure to take account of the importance of population context, as an effect modifier and determinant of individual level exposures… the major population determinants of health are ignored and undue attention is focused on individual characteristics” [35].

5 ‘Human Ecology’, a term coined by Ellen Swallow Richards in 1907, suggests a holistic approach to the exploration of the spatial and temporal interrelationships between humans and their natural, economic, social and built environments and political organization, or more succinctly, man’s collective interaction with his environment.
